# A Novel Cys2His2 Zinc Finger Homolog of AZF1 Modulates Holocellulase Expression in *Trichoderma reesei*

**DOI:** 10.1128/mSystems.00161-19

**Published:** 2019-06-18

**Authors:** Amanda Cristina Campos Antonieto, Karoline Maria Vieira Nogueira, Renato Graciano de Paula, Luísa Czamanski Nora, Murilo Henrique Anzolini Cassiano, Maria-Eugenia Guazzaroni, Fausto Almeida, Thiago Aparecido da Silva, Laure Nicolas Annick Ries, Leandro Jose de Assis, Gustavo Henrique Goldman, Roberto Nascimento Silva, Rafael Silva-Rocha

**Affiliations:** a FMRP—University of São Paulo, Ribeirão Preto, SP, Brazil; b FFCLRP—University of São Paulo, Ribeirão Preto, SP, Brazil; c FCFRP—University of São Paulo, Ribeirão Preto, SP, Brazil; d Institute for Advanced Study, Technical University of Munich, Garching, Germany; University of Queensland

**Keywords:** *cis*-regulatory elements, holocellulase expression, regulatory networks, systems biology

## Abstract

Filamentous fungi are remarkable producers of enzymes dedicated to the degradation of sugar polymers found in the plant cell wall. Here, we integrated transcriptomic data to identify novel transcription factors (TFs) related to the control of gene expression of lignocellulosic hydrolases in Trichoderma reesei and Aspergillus nidulans. Using various sets of differentially expressed genes, we identified some putative *cis*-regulatory elements that were related to known binding sites for Saccharomyces cerevisiae TFs. Comparative genomics allowed the identification of six transcriptional factors in filamentous fungi that have corresponding S. cerevisiae homologs. Additionally, a knockout strain of T. reesei lacking one of these TFs (S. cerevisiae *AZF1* homolog) displayed strong reductions in the levels of expression of several cellulase-encoding genes in response to both Avicel and sugarcane bagasse, revealing a new player in the complex regulatory network operating in filamentous fungi during plant biomass degradation. Finally, RNA sequencing (RNA-seq) analysis showed the scope of the AZF1 homologue in regulating a number of processes in T. reesei, and chromatin immunoprecipitation-quantitative PCR (ChIP-qPCR) provided evidence for the direct interaction of this TF in the promoter regions of *cel7a*, *cel45a*, and *swo*. Therefore, we identified here a novel TF which plays a positive effect in the expression of cellulase-encoding genes in T. reesei.

**IMPORTANCE** In this work, we used a systems biology approach to map new regulatory interactions in Trichoderma reesei controlling the expression of genes encoding cellulase and hemicellulase. By integrating transcriptomics related to complex biomass degradation, we were able to identify a novel transcriptional regulator which is able to activate the expression of these genes in response to two different cellulose sources. *In vivo* experimental validation confirmed the role of this new regulator in several other processes related to carbon source utilization and nutrient transport. Therefore, this work revealed novel forms of regulatory interaction in this model system for plant biomass deconstruction and also represented a new approach that could be easy applied to other organisms.

## Abstract (precis)

In this work, we used a systems biology approach to map new regulatory interactions in Trichoderma reesei controlling the expression of genes encoding cellulase and hemicellulase. By integrating transcriptomics related to complex biomass degradation, we were able to identify a novel transcriptional regulator which is able to activate the expression of these genes in response to two different cellulose sources. *In vivo* experimental validation confirmed the role of this new regulator in several other processes related to carbon source utilization and nutrient transport. Therefore, this work revealed novel forms of regulatory interaction in this model system for plant biomass deconstruction and also represented a new approach that could be easy applied to other organisms.

## INTRODUCTION

Plant cell walls are the most abundant source of polysaccharides and are formed by a complex mix of sugar polymers that are highly recalcitrant to degradation. This abundant biomass has been targeted in the past years as a source of building blocks for both the production of fine chemicals and the generation of biofuels from renewable sources (1). Accordingly, a special emphasis has been placed on obtaining and engineering biotechnologically relevant hydrolytic enzymes with enhanced performance for plant biomass degradation, where endoglucanases (EC 3.2.1.4), exoglucanases (EC 3.2.1.91), and β-glucosidases (EC 3.2.1.21) are the major players (2, 3). Filamentous fungi are key players in the process of degradation of plant biomass in natural environments, and organisms from several groups are endowed with a tremendous portfolio of hydrolytic enzymes capable of decomposing the complex polysaccharides (cellulose, hemicellulose, and pectin) and lignin into their basic sugar and alcohol components (4, 5). Enzymes from these organisms are already being used in industrial processes. Together with this arsenal of enzymes, organisms from this group have evolved a sophisticated machinery to sense the presence of these complex substrates and control the expression of these hydrolytic enzymes in response to their presence (5, 6). In this sense, the regulatory network controlling the expression of cellulases and hemicellulases has been extensively investigated at the molecular level in a number of filamentous fungi, such as *Trichoderma* spp., *Aspergillus* spp., Neurospora crassa, and *Penicillium* spp., among others (5, 6). Decades of investigation have revealed a number of transcription factors (TFs) that control the expression of genes encoding hydrolytic enzymes (5, 7). Two remarkable features uncovered in these studies are that the repertoires of cellulase/hemicellulase-encoding genes differ in different fungal genomes and the regulatory networks in the organisms have different architectures and are formed by both homologous TFs and genus-specific TFs. In this particular regard, expression of hydrolytic enzymes is usually dependent on the presence of a XlnR/XYR1 transactivator (8) when cells are exposed to lignocellulosic components, while the repression mediated by CreA/CRE1 regulator occurs when glucose is present in the medium (9). In fact, these carbon catabolite repression (CCR) mechanisms operate both directly and indirectly, as XlnR/XYR1 homologs are usually blocked by CreA/CRE1 (10). In addition to these master regulators, a number of specific TFs have been identified in several organisms as modulators of expression of hydrolytic enzymes in response to subcomponents generated during lignocellulosic material degradation (5). For instance, CRL-1 and CRL-2 have been described as the major activators of cellulase-encoding genes in N. crassa (11), while activation of hydrolytic enzymes in Trichoderma reesei has been reported to be mediated by several proteins such as ACE2, BglR, and ACE3 and to be facilitated by the HAP2/3/5 complex as well as the Lae1 methyltransferase (12). However, in the case of T. reesei, proteins ACE1 and Xpp1 have been described as repressors of certain hydrolytic enzymes (13, 14), indicating that a complex regulatory network operates in this organism which is not yet completely understood. As T. reesei is a major cellulase/hemicellulase-producing fungus, deciphering its intricate regulatory network for enzyme production should strongly facilitate its genetic engineering for biotechnologically relevant applications.

In recent years, the global response of filamentous fungi to complex lignocellulosic material has been widely investigated (4, 15–19). Therefore, the combination of transcriptomic and proteomic approaches has provided useful information on the regulatory scope of many of the known transcriptional regulators during biomass exposure. These high-throughput techniques have revealed a number of regulons for which the fine molecular mechanisms are not yet known (4, 17, 20–22). Here, we integrated high-throughput data with comparative genomics to discover novel regulatory elements related to the expression of genes encoding cellulases and hemicellulases in T. reesei and in the model organism Aspergillus nidulans. Through this approach, we identified a number of putative *cis*-regulatory elements within sets of promoters of genes related to TFs and hydrolase-encoding genes that are differentially expressed under several growth conditions. A comparison of these elements with those previously characterized in Saccharomyces cerevisiae has led to the identification of six putative TFs involved in the regulation of such genes under different growth conditions. From these candidates, gene disruption of a Cys2His2 zinc finger homolog of S. cerevisiae *AZF1* in T. reesei revealed that this TF was directly involved in the modulation of cellulase/hemicellulase expression in the fungus. Taking the data together, this work adds a new player to the intricate regulatory network controlling hydrolytic enzyme production in a biotechnologically relevant organism that can become a new target for genetic engineering of strains for industrial application.

## RESULTS

### Identification of candidate *cis*-regulatory elements using transcriptomic data from T. reesei and A. nidulans.

**FIG 1:**
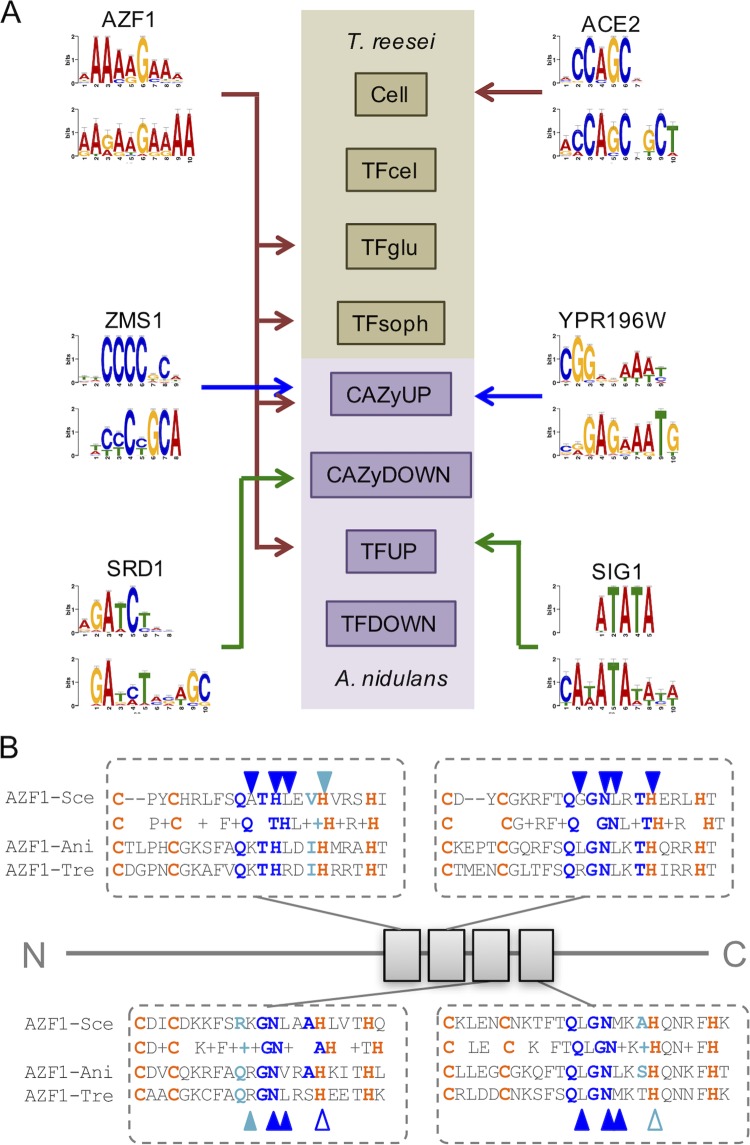
Mining novel *cis*-regulatory elements in transcriptomic data of filamentous fungi. (A) Eight sets of differentially expressed genes from T. reesei and A. nidulans high-throughput experiments were used for *de novo* identification of putative *cis*-regulatory elements. Six revealed DNA motifs with similarity to binding sites characterized in S. cerevisiae (in the comparison, the upper part represents the motif in S. cerevisiae, while the lower part represents the identified motif). From these, AZF1-like homologs were found to be overrepresented under two or more sets of conditions and in both T. reesei and A. nidulans. (B) Comparison between AZF1 homologs in T. reesei (Tre) and A. nidulans (Ani) at the four Cys2His2 zinc fingers. Orange highlights the conserved Cys and His amino acids for zinc binding; dark blue represents positions known to make sequence specific contacts in the target DNA sequence. Open symbols mark positions less highly conserved between the three homologs. Sce, S. cerevisiae.

In order to identify novel TFs controlling cellulase/hemicellulase expression in filamentous fungi, we investigated high-throughput data from T. reesei exposed to cellulose, sophorose (a disaccharide that is produced via the activity of exoglucanases and is known to act as a potent inducer of cellulase in T. reesei), and glucose (17) and from A. nidulans exposed to sugarcane bagasse (SCB) (23). We anticipated that if we could identify candidate homologues conserved in both organisms, they would have higher levels of changes in functional TFs. Next, we selected hydrolytic enzymes or TFs differentially expressed under each set of conditions and performed *de novo* DNA motif identification in the region 1,000 bp upstream of the genes of interest. The data used for motif discovery were divided into four groups of differentially expressed genes from T. reesei (24) and four groups of TFs or carbohydrate-active enzymes (CAZy)-related genes upregulated or downregulated in A. nidulans exposed to sugarcane bagasse (23). For each data set, we selected the top 10 candidate motifs and performed peer-to-peer comparisons of each motif within and between the two organisms. Nonnegative matrix factorization analysis of the comparison revealed a number of motifs shared under different conditions and between the two organisms (see Fig. S1 in the supplemental material). Next, from the total 80 motifs identified, we compared 1 to the database of *cis*-regulatory elements characterized in S. cerevisiae (25). Using this approach, we identified nine candidate motifs that were similar to *cis* elements from yeast with a level of high confidence (*P* < 0.0001), representing the potential binding sites for six different TFs from S. cerevisiae (Fig. 1A). Each of the horizontal rectangles in Fig. 1 represents the set of genes analyzed for each organism (four set for T. reesei and four for A. nidulans), and the arrows indicate the *cis* elements that were enriched in the indicated data set. Subsequently, one motif with similarity to the AZF1-binding site was of particular interest because it was enriched in the promoter region of TFs upregulated in the presence of glucose and sophorose in T. reesei and in the promoter regions of TFs and of CAZy-encoding genes in A. nidulans, indicating that it could represent the binding site for a conserved regulator of hydrolytic enzymes expressed in both organisms. When we used the predicted binding sites for the six putative TFs to map potential target promoters at the genome scale in T. reesei and A. nidulans, we observed that putative AZF1 binding sites were more enriched in natural promoters than in randomly generated sequences (Fig. S2). This result suggests a potential role of this *cis* element as a target of a TF in filamentous fungi.

10.1128/mSystems.00161-19.1 FIG S1 Comparison of identified motifs using nonnegative matrix factorization (NNMF). For each of the eight data sets, 10 motifs were predicted using MEME. Next, peer-to-peer comparison was performed with TOMTOM, generating an 80-by-80 matrix. Next, data were loaded in MeV (http://mev.tm4.org/) and analyzed using nonnegative matrix factorization. The left side shows examples of motifs clustered together. Download FIG S1, PDF file, 0.6 MB. Copyright © 2019 Antonieto et al. 2019 Antonieto et al. This content is distributed under the terms of the Creative Commons Attribution 4.0 International license.

10.1128/mSystems.00161-19.2 FIG S2 Mapping of putative binding sites for the *cis*-regulatory elements in the genomes of T. reesei and A. nidulans. For motif searches, a 1,000-bp upstream region from each gene annotated for both organisms was used. The putative DNA motifs identified in Fig. 1A were mapped in the promoters of T. reesei (AZF1 and ACE2) and A. nidulans (ZMS1, YPR196W, SIG1, and SRD1). The score distributions in the real promoter datasets are represented in blue, while scores found in a control random sequence dataset are represented in yellow. Download FIG S2, PDF file, 0.4 MB. Copyright © 2019 Antonieto et al. 2019 Antonieto et al. This content is distributed under the terms of the Creative Commons Attribution 4.0 International license.

**FIG 2:**
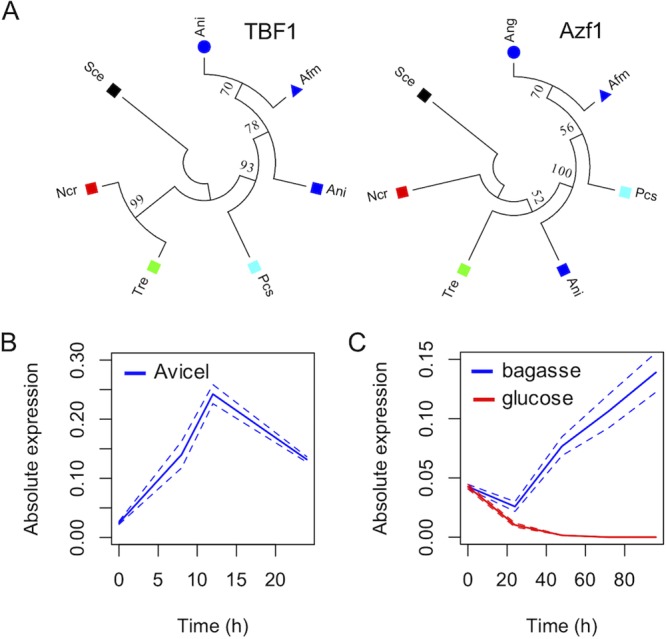
Characterization of AZF1 homolog in T. reesei. (A) Phylogenetic tree representing the relationship of AZF1 homologs found in the genome of six filamentous fungi compared to a TBF1 housekeeping protein. This analysis indicates that the evolutionary history of AZF1 homologs in filamentous fungi does not perfectly match the history of the studied group. (B) Expression profile of TrAZF1 in Avicel analyzed using real-time PCR. (C) Expression profile of TrAZF1 in sugarcane bagasse and glucose.

We next used the protein sequence of AZF1 of S. cerevisiae to identify the homologs in T. reesei and A. nidulans, leading to the identification of the protein 103275 in the former and the protein AN6503 in the later. These two proteins share approximately 37% identity with the homolog in S. cerevisiae and are annotated as hypothetical proteins. The first remarkable change in these homologs is that while *AZF1* from yeast has 914 amino acids, the proteins found in the filamentous fungi are half the size (506 amino acids in T. reesei and 457 amino acids in A. nidulans). Sequence comparison between the three proteins led to the identification of four conserved Cys2His2-type zinc fingers, with 13 of 16 critical amino acids known to make specific interactions with DNA being conserved in these domains (Fig. 1B). These analyses indicate that such *AZF1* homologs are conserved at least at the DNA-binding domains and that they could be functional in the filamentous fungi analyzed. Phylogenic analysis of the *AZF1* homologs in several filamentous fungi indicates that the evolutionary history of these regulators does not follow exactly that of a housekeeping TF such as TBF1 (Fig. 2A; see also Fig. S3), reinforcing the notion that divergence in these protein sequences has occurred during the evolution of this group, which may have led to the rise of novel regulatory functions.

10.1128/mSystems.00161-19.3 FIG S3 Phylogenetic tree of orthologous TFs from filamentous fungi. For each homologue, the protein sequence found in the genome of S. cerevisiae, T. reesei, N. crassa, Penicillium chrysogenum, A. nidulans, A. niger, and A. fumigatus. For comparison, the phylogeny of a housekeeping transcription factor (TBF1) is presented. For clarity, each group is represented by a specific color. Phylogenetic trees were constructed using the neighbor joining method in Mega 6.0 software (https://www.megasoftware.net/). Download FIG S3, PDF file, 0.1 MB. Copyright © 2019 Antonieto et al. 2019 Antonieto et al. This content is distributed under the terms of the Creative Commons Attribution 4.0 International license.

### An AZF1 homolog from T. reesei is induced by plant biomass and repressed by glucose.

In order to assess the role of *AZF1* in T. reesei, we first quantified the expression of this regulatory gene in the presence of different carbon sources. We found that expression of this gene was strongly induced in media supplemented with cellulase (Avicel) and in pretreated SCB, while the level of expression in glucose was virtually undetectable (Fig. 2B and C). This strong induction observed during exposure to plant biomass material and repression observed with glucose as a carbon source indicate that this TF could be implicated in the modulation of gene expression of hydrolytic enzymes in response to complex substrates. In order to further investigate the function of the AZF1 protein in T. reesei, we constructed a deletion mutant strain using homologous recombination (Fig. S4A). This mutant showed normal growth in peptone-dextrose agar (PDA) medium but showed some growth defects during growth on arabinose (Fig. S4B) as well as sensitivity to extreme pH variations (Fig. S4C). While the S. cerevisiae *AZF1* gene is involved in the control of carbohydrate utilization pathways and the cell cycle during growth in glucose, T. reesei lacking *AZF1* displayed a delay in spore formation as well as a slight reduction in spore size (Fig. S5). Also, AZF1 from S. cerevisiae is a glucose-dependent transcriptional activator (26), and the homologue from T. reesei was repressed by glucose and induced by SCB. Therefore, we named the product of this gene TrAZF1 (for “T. reesei AZF1”).

10.1128/mSystems.00161-19.4 FIG S4 Deletion strategy and confirmation of the mutant Δ*azf1*. (A) The construction of the mutant strain was performed by replacing the open reading frame (ORF) of the *azf1* gene (Tr_103275) with that of the hygromycin resistance gene (*hph*). Southern blot analysis resulted in two fragments of 6.2 kb and 2.16 kb, corresponding to the parental and mutant strains, respectively. (B) Growth of the Δ*azf1* mutant in different carbon sources. Totals of 10^6^ spores/ml of the parental and mutant strains were grown in deep-well plates containing 5 ml of Mandels-Andreotti medium with 1% of each carbon source for 24 h. The mycelium were collected by vacuum filtration and dried for 90 min at 75°C. Statistically significant differences between the parental and mutant strains were observed only for arabinose (***, *P* < 0.001). (C) Deletion of *azf1* can affect fungal growth in response to ambient pH. The TU6_Δ*tku70* and Δ*azf1* strains were cultivated on PDA plates with pH adjusted to 5.0, 7.0, 9.0, and 11.0. The incubation was carried out at 28°C, and growth was registered in days 4 and 7. Download FIG S4, PDF file, 0.2 MB. Copyright © 2019 Antonieto et al. 2019 Antonieto et al. This content is distributed under the terms of the Creative Commons Attribution 4.0 International license.

10.1128/mSystems.00161-19.5 FIG S5 AZF1 is involved in sporulation mechanism in T. reesei. Flow cytometry analysis was performed with the TU6_Δ*tku70* (A) and Δ*azf1* (B) spores after 7 days of growth in MEX medium. Cell size and complexity were detected by the use of a forward scatter detector (FSC-H) and side scatter detector (SSC-H), respectively. (C) The growth of the TU6_Δ*tku70* and Δ*azf1* strains in race tubes containing MEX medium was evaluated for a period of 7 days. Download FIG S5, PDF file, 0.1 MB. Copyright © 2019 Antonieto et al. 2019 Antonieto et al. This content is distributed under the terms of the Creative Commons Attribution 4.0 International license.

### TrAZF1 positively modulates cellulase gene expression in response to plant biomass material.

**FIG 3:**
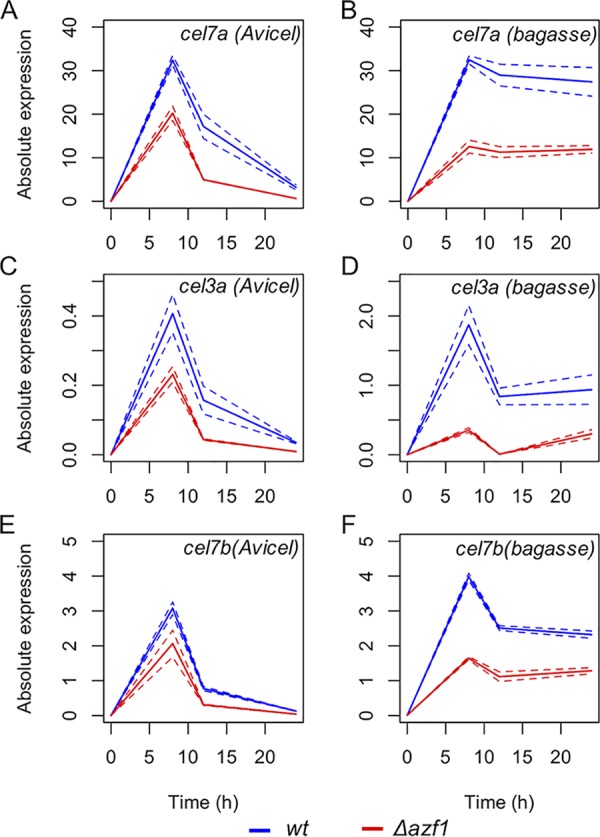
Effect of TrAZF1 on the expression of holocellulase-encoding genes. Wild-type (wt) and Δ*azf1* mutant strains of T. reesei were induced with cellulose (Avicel; A, C, and E) or sugarcane bagasse (bagasse; B, D, and F) after growing in liquid media with glycerol. Samples were taken 8, 12, and 24 h after induction, and gene expression was assessed by real-time PCR. Solid lines represent the mean levels of expression measured in three independent experiments, and dashed lines represent the upper and lower limits of standard deviation. The genes assessed were *cel7a* (A and B), *cel3a* (C and D), and *cel7b* (E and F).

**FIG 4:**
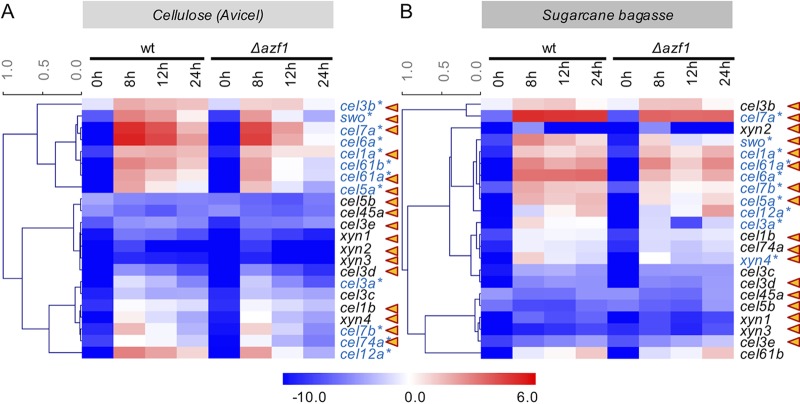
Cellulolytic and hemicellulolytic genes are under AZF1 control in T. reesei. Gene expression of 17 cellulases, 4 xylanases, and swollenin protein was evaluated after cultivation of the T. reesei TU6_Δ*tku70* (wild-type) strain and the T. reesei TU6_Δt*ku70*Δ*azf1* (Δ*azf1*) strain in the presence of cellulose and sugarcane bagasse for 8, 12, and 24 h. MeV 4.8.1 software was used for heat map construction. Gene expression values represent means of results from three independent experiments expressed in log2. Triangles in orange represent promoters with potential binding sites for TrAZF1. The genes indicated in blue were differentially expressed between the wild-type and Δ*azf1* strains. Asterisks indicate differentially expressed genes.

To assess the potential function of TrAZF1 in the modulation of expression of hydrolytic enzymes, we searched the promoters of a set of 22 candidate cellulases/hemicellulases from T. reesei (see Table S1 in the supplemental material) for potential binding sites for this regulator. From this, we found 17 promoters with potential binding sites with scores ranging from 11.7 to 14.0 (Table S2). In order to experimentally assess the role of TrAZF1 in the expression of these genes, we performed real-time quantitative PCR (RT-qPCR) for the 22 candidates in wild-type and Δ*azf1* strains exposed to Avicel and pretreated sugarcane bagasse (SCB). The data presented in Fig. 3 show that the levels of expression of *cel7a*, *cel3a*, and *cel7b* were significantly reduced in the mutant strain of TrAZF1 when induced with either cellulose or SCB. Yet the analysis of 22 holocellulase-encoding genes demonstrated that most of them were downregulated in the mutant strain of TrAZF1 under the two sets of inducing conditions tested (Fig. 4). Binding site identification shows that 7 of the 12 genes differentially expressed in the mutant strain under cellulose growth conditions had a putative TrAZF1 binding site (Table S2), suggesting that this regulator in fact participates in the modulation of cellulase/hemicellulase-encoding genes in T. reesei.

10.1128/mSystems.00161-19.8 TABLE S1 Datasets and primers used for motif prediction. Download Table S1, DOCX file, 0.02 MB. Copyright © 2019 Antonieto et al. 2019 Antonieto et al. This content is distributed under the terms of the Creative Commons Attribution 4.0 International license.

10.1128/mSystems.00161-19.9 TABLE S2 Prediction of binding sites in holocellulase-encoding genes. Download Table S2, DOCX file, 0.01 MB. Copyright © 2019 Antonieto et al. 2019 Antonieto et al. This content is distributed under the terms of the Creative Commons Attribution 4.0 International license.

### TrAZF1 regulates several categories of genes related to the biomass degradation.

**FIG 5:**
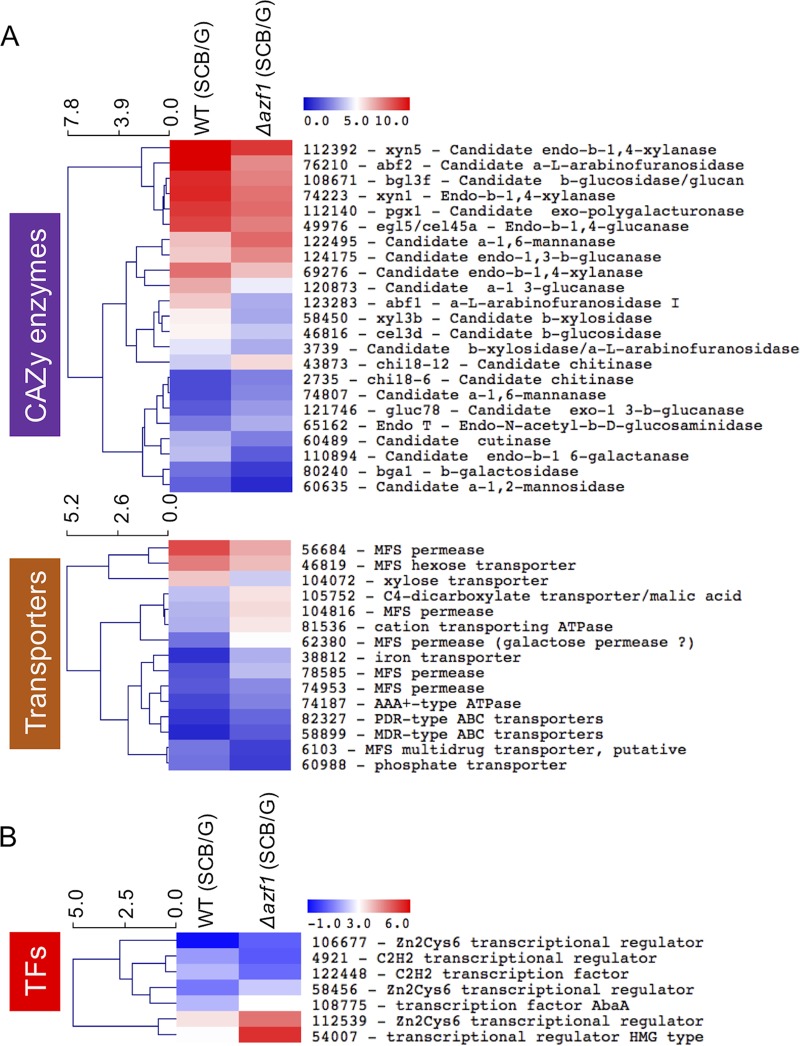
Heat maps of differentially expressed genes determined by RNA-seq upon induction with SCB. The heat maps are related to the CAZy (A [top panel]) and transporter (A [bottom panel]) categories and to the TF category (B). Expression values represent log2-fold change between strains grown in SCB normalized to strains grown in glycerol, and colors are according to the scale in the figure.

**FIG 6:**
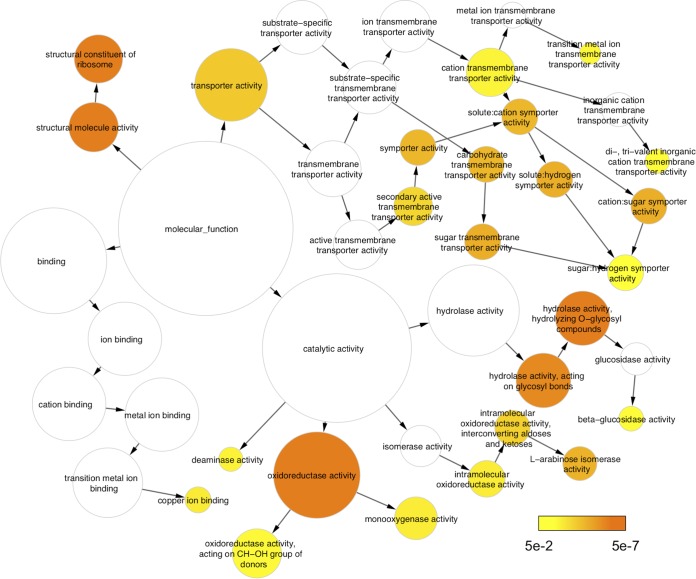
Gene Ontology analysis of the TrAZF1-modulated genes. Genes belonging to the main category “molecular function” that were found to be enriched in the analysis and the corresponding GO terms are indicated. The GO network was analyzed in Cytoscape using BINGO (38).

A global analysis of the transcriptome of T. reesei wild-type and Δ*azf1* strains grown in SCB or glucose was performed to verify which genes are under the control of the TrAZF1 transcription factor during the degradation of the plant biomass. The T. reesei genome comprises 9,129 genes, and 2,088 genes were differentially expressed in one or more of the comparisons performed (Table S3; see also Fig. S6). A total of 347 genes were differentially expressed between the wild-type and Δ*azf1* strains grown in SCB. The data shown in Fig. 5 highlight three groups of genes encoding CAZymes (23 genes; Fig. 5A, top panel), transporters (15 genes; Fig. 5A, bottom panel), and TFs (7 genes; Fig. 5B). Two genes encoding α-l-arabinofuranosidases, *abf2* and *abf1* (identifiers [ID] 76210 and 123283, respectively), were the CAZyme-encoding genes that were most highly induced by TrAZF1. Both showed around a 7-fold reduction in expression in the Δ*azf1* mutant strain in comparison to the wild-type strain, followed by genes encoding a candidate β-xylosidase (ID 58450), a candidate α-1,3-glucanase (ID 120873), and a candidate endo-β-1,4-xylanase, *xyn5* (ID 112392). Cellulase genes *cel3d* (ID 46816) and *cel45a* (ID 49976) are also regulated by the TrAZF1 transcription factor, exhibiting a 2-fold reduction in expression in the Δ*azf1* strain. In the case of transporters, the major transporter positively regulated by TrAZF1 is the xylose transporter (ID 104072), exhibiting around a 4-fold reduction in expression in the Δ*azf1* mutant strain compared to the parent strain. The MFS permeases are responsible for more than half of the transporter genes regulated by TrAZF1. The MFS permease (ID 56684) and the MFS hexose transporter (ID 46819) genes are the MFS genes most highly induced by TrAZF1, while the MFS permease (ID 62380), a galactose permease, showed around a 7-fold reduction in expression in the wild-type background compared to the mutant strain. Finally, among the 7 TFs with differential expression in the mutant strain, two genes (ID 122448 and ID 4921) encode C2H2 transcriptional regulators. The other five genes were repressed by TrAZF1. The most highly repressed genes encode a transcriptional regulator HMG-type (ID 54007) and showed 6-fold higher expression in the mutant Δ*azf1* strain than in the parental strain. Additionally, the analysis of the top 15 potentially TrAZF1-activated genes demonstrated enrichment for CAZy enzymes, while the list of highly repressed genes is more diverse in terms of functional categories (Fig. S6C). An enrichment analysis using the terms of Gene Ontology (GO) showed that TrAZF1-modulated genes belonging to the “molecular function” category have their functions mainly related to “transporter activity” and “oxidoreductase activity” (Fig. 6). Among the genes related to the “cellular component,” the main enriched GO terms are related to “membrane” (Fig. S7A), and in the “biological process” category, most of the genes included are related to “carbohydrate metabolic process” (Fig. S4C). Taken together, these data reinforce the idea of a role of TrAZF1 in the regulation of plant biomass degradation.

10.1128/mSystems.00161-19.6 FIG S6 Analysis of RNA-seq differential expression data quality. After performing differential expression analysis, we used a hierarchical clustering heat map to obtain an overview of the differences and similarities of gene expression from each samples based on sample distance. We determined that the samples from each of triplicate instances of the experimental design (i.e., a strain examined under growth conditions) showed similar expression patterns. (B) Principal-component analysis (PCA) of the differential gene expression. By reducing the dimensionality by using the 2 principal components, we show the differentiation in expression patterns between the two growth conditions (i.e., libraries from SCB are on the left, while those from glycerol are on the right, and differential results from comparisons between wild-type and mutant strains are presented with wild-type libraries shown on the top and mutant libraries on the bottom). We also show that the expression patterns were similar in the triplicate experiments. (C) Heat map of top 15 deferentially expressed genes determined by RNA-seq upon induction with SCB. The most highly induced genes are represented at the top, while the most highly repressed genes are represented at the bottom. Expression values represent log2-fold changes between SCB results normalized to strains grown in glycerol, and colors correspond to the scale in the figure. Download FIG S6, PDF file, 0.1 MB. Copyright © 2019 Antonieto et al. 2019 Antonieto et al. This content is distributed under the terms of the Creative Commons Attribution 4.0 International license.

10.1128/mSystems.00161-19.7 FIG S7 Gene Ontology analysis of the TrAZF1-modulated genes. (A) Genes belonging to the main category “cellular component” and its respective GO terms enriched in the analysis. (B) Genes belonging to the main category “metabolic process” and its respective GO terms enriched in the analysis. GO network analyses were performed in Cytoscape using BINGO. Download FIG S7, PDF file, 0.08 MB. Copyright © 2019 Antonieto et al. 2019 Antonieto et al. This content is distributed under the terms of the Creative Commons Attribution 4.0 International license.

10.1128/mSystems.00161-19.10 TABLE S3 Differentially expressed genes. Download Table S3, XLSX file, 0.04 MB. Copyright © 2019 Antonieto et al. 2019 Antonieto et al. This content is distributed under the terms of the Creative Commons Attribution 4.0 International license.

### Cellulase activity is decreased in the Δ*azf1* mutant.

**FIG 7:**
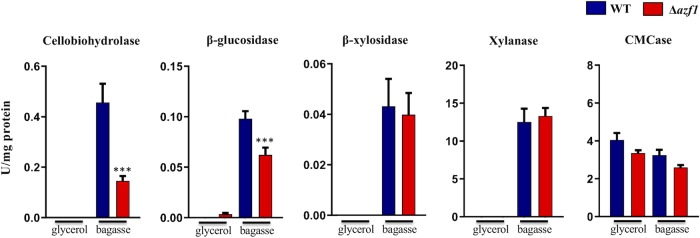
Cellulolytic and hemicellulolytic activity during growth of the T. reesei wild-type (WT) and Δ*azf1* strains in sugarcane bagasse. Levels of cellobiohydrolase, β-glucosidase, β-xylosidase, xylanase, and CMCase activity were measured after 24 h of growth in sugarcane bagasse, pregrown in glycerol. Error bars indicate standard deviations of results from the triplicate experiments (*, *P* < 0.05; **, *P* < 0.01, ***, *P* < 0.001).

Cellulase and hemicellulase activities were tested in the parental and Δ*azf1* strains after 24 h of growth in sugarcane bagasse, after previous growth in glycerol. Decreased secretion of β-glucosidases and cellobiohydrolases was observed in the Δ*azf1* strain compared to the parental strain in sugarcane bagasse, suggesting that TrAZF1 could be related to the positive regulation of cellulase production. No significant difference was observed for the levels of xylanase and β-xylosidase activity between the parental and mutant strains grown in sugarcane bagasse. As expected, no enzymatic activity was observed for either of the strains during growth in glycerol (as the sole carbon source), except for carboxymethylcellulase (CMCase), for which a low level of activity could be detected in both strains (Fig. 7). These results confirmed that a lack of TrAZF1 not only influenced the expression level of cellulase-encoding genes, as demonstrated by real-time PCR (RT-PCR) and transcriptome sequencing (RNA-seq) analyses, but also caused an overall reduction in the level of production of the main cellulase by this fungus.

### *TrAZF1* directly binds to the promoter region of *cel7a*, *cel45a*, and *swo*.

**FIG 8:**
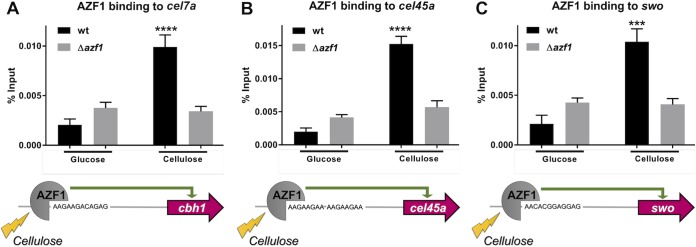
AZF1 binding to the promoter region of *cel7a*, *cel45a*, and *swo*. Chromatin immunoprecipitation coupled to qPCR was performed with WT and Δ*azf1* strains grown in the presence of glucose and cellulose (*, *P* < 0.05; **, *P* < 0.01; ***, *P* < 0.001; ****, *P* < 0.0001).

In order to investigate whether the effect of TrAZF1 on the target genes was direct or indirect, we performed chromatin immunoprecipitation followed by quantitative PCR (ChIP-qPCR) in wild-type and mutant strains of T. reesei for three target genes. For this, a monoclonal antibody against purified recombinant TrAZF1 protein was used to immunoprecipitate the protein-DNA complex after cross-linking performed as described before (27). Next, three primer pairs targeting the identified putative AZF1-binding region at the *cel7a*, *cel45a*, and *swo* promoter regions (Fig. 8) were used to quantify the precipitated fragments by real-time PCR. As shown in Fig. 8, we found no difference for any of the tested promoters when the wild-type and mutant strains of T. reesei were grown in the presence of glucose as the carbon source. However, when the clones were grown in Avicel, we found a strong increase in the level of DNA fragment only in wild-type strain, indicating that TrAZF1 was strongly associated with the target promoters when cells were induced with Avicel. Taken together, these results reinforce the notion that TrAZF1 acts as an activator of gene expression through the direct interaction with the target promoters.

## DISCUSSION

We present here the integration of high-throughput data with comparative genomics to identify novel potential TFs related to the control of hydrolytic enzyme-encoding genes in filamentous fungi, with special focus on T. reesei and A. nidulans. The use of this integrative approach allowed the identification of a number of putative TFs, which led to the characterization of an AZF1 homolog in T. reesei and evidenced the discovery of a novel Cys2His2-type zinc finger regulator able to modulate the expression of hydrolytic enzyme-encoding genes in response to plant biomass material sources. It is quite interesting not only that the protein arrangement had been modified during the evolution of the homologous proteins (evidenced by a strike reduction in protein size) but also that their regulation and functional scope have been drastically reshaped, at least in T. reesei. While the AZF1 from S. cerevisiae is a glucose-dependent positive regulator whose level of expression is constant in different carbon sources (26, 28), TrAZF1 acts as a positive regulator upon exposure to plant biomass material, and the evidence provided here indicates that it directly interacts with its target promoters. The expression is completely repressed in the presence of glucose. This new regulatory link implies that TrAZF1 is also subjected to CCR in a way similar to that previously reported for XYR1 (10), the master cellulase regulator in T. reesei. This fact implies that while it is necessary for enhanced expression of hydrolytic enzyme-encoding genes, TrAZF1 is still placed downstream in the regulatory hierarchy controlling carbohydrate utilization in filamentous fungi (as it is subject to CCR). Yet this novel regulator, as well as its homologs found in the genomes of other filamentous fungi, could serve as a potential target(s) in the future for genetic engineering of strains with enhanced cellulase/hemicellulase production for biotechnological applications. To the best of our knowledge, this is the first time that an AZF1 homolog has been described that modulates the expression of cellulase-encoding genes in a filamentous fungus. As this regulator was conserved between T. reesei and A. nidulans, this would indicate that the mechanisms investigated here could also occur in the latter, but this should be further investigated in future studies. Furthermore, additional analysis will be necessary to demonstrate the extent to which the homologs of this regulator that are present in the genome of other filamentous fungi can also participate in the regulation of cellulase production under inducing conditions. Finally, the approach presented here demonstrates the potential of a computational approach to integrate high-throughput data to discover novel TFs controlling complex networks in organisms of interest.

## MATERIALS AND METHODS

### Strains and growth conditions.

T. reesei strains QM9414 (ATCC 26921), TU6_Δ*tku70*, and Δ*azf1* were grown in MEX medium (3% [wt/vol] malt extract, 0.1% [wt/vol] peptone, 1.5% [wt/vol] agar) at 28°C for a period of 7 days. To verify the expression of *azf1* in sugarcane bagasse and glucose, 10^6^ spores of QM9414 were inoculated into 200 ml of Mandels-Andreotti medium (29) containing 1% glycerol; after 24 h, the mycelium was transferred into 200 ml of Mandels-Andreotti medium containing a 1% concentration of sugarcane bagasse or a 2% concentration of glucose. The flasks were incubated in an orbital shaker at 200 rpm and 28°C, and the mycelium was collected at 24, 48, 72, and 96 h for sugarcane bagasse and at 24 and 48 h in the case of glucose. For induction experiments, 10^6^ spores of the TU6_Δ*tku70* and Δ*azf1* strains were inoculated in 25 ml of Mandels-Andreotti medium containing 1% glycerol. After 24 h, the mycelium was collected, washed with Mandels-Andreotti medium without a carbon source, and transferred to 25 ml of Mandels-Andreotti medium with 1% cellulose or 1% sugarcane bagasse. The flasks were incubated in an orbital shaker at 200 rpm and 28°C for 8, 12, and 24 h. For the TU6_Δ*tku70* and Δ*azf1* strains, 10 mM uridine was added to all cultivation media. The experiments were conducted in triplicate for each sample. After induction, the mycelia were collected by filtration, frozen, and stored at −80°C.

### Discovery and analysis of DNA motifs.

For identification of overrepresented DNA motifs, eight data sets of differentially expressed genes and several growth conditions were used. The first four data sets were groups of genes used to identify the putative binding site for XYR1 and CRE1 regulators in T. reesei (24). In the list of 22 hydrolytic enzyme-encoding genes, 7 TFs were upregulated under conditions of Avicel growth, 18 TFs were upregulated under conditions of growth in glucose, and 18 TFs were upregulated under conditions of growth in Sophorose (24). Additionally, we used four data sets corresponding to differently expressed genes from A. nidulans exposed to sugarcane bagasse, with 54 TF genes that were upregulated and 18 TF genes that were downregulated, along with 88 CAZy genes that were upregulated and 28 CAZy genes that were downregulated under these conditions. The list of genes used in each data set is presented in Table S1 in the supplemental material. For each data set, 1,000 bp upstream sequences representing the promoter region were used for *de novo* motif discovery using MEME (30). Search parameters were set to identify short (6-bp to 10-bp) unique elements that were overrepresented in the data sets. The sets of identified motifs were compared to entries in the JASPAR database of *cis*-regulatory elements in Saccharomyces cerevisiae by the use of TOMTOM, to identify similar motifs related to previously characterized elements. Finally, the identified elements were mapped into the corresponding genome sequences using *ad hoc Perl* scripts.

### Construction of the Δ*azf1* mutant strain.

The methodology for obtaining the mutant was adapted from a protocol described previously (31), wherein the deletion cassette is obtained by homologous recombination in yeast. The primers used for amplification of hygromycin and the promoter and terminator regions of the *Tr*_103275 gene were selected according to a previously described protocol (31) and are listed in Table S1. The selection marker used was the hygromycin resistance gene (*hph*, encoding Escherichia coli hygromycin B phosphotransferase). The fungus used for the transformation was the T. reesei TU6_Δ*tku70* parental strain, which has uridine auxotrophy. Gene deletion was confirmed with Southern blotting using a digoxigenin (DIG)-High Prime DNA labeling and detection starter kit (Roche) according to the manufacturer's instructions.

### Evaluation of growth and sporulation profile of the Δ*azf1* mutant.

To evaluate the growth profile of the Δ*azf1* mutant in different carbon sources, 10^6^ spores/ml of the parental and mutant strains were inoculated in deep-well plates containing 5 ml of Mandels-Andreotti medium with 1% of each carbon source (maltose, lactose, arabinose, mannose, glucose, starch, xylose, and glycerol). The plates were incubated at 30°C and 180 rpm for 24 h. The mycelia were collected by vacuum filtration and dried for 90 min at 75°C. All the experiments were performed in triplicate. Growth at different pH values was also evaluated. For this purpose, plates with PDA medium (pH 5.6) were adjusted to pH 5, 7, 9, and 11 with either 3 M HCl or 6 M NaOH. The growth on the plates was checked after 4 and 7 days. The sporulation profile of parental and mutant strains in race tubes was done with MEX medium, and the growth was observed for 14 days.

### Flow cytometry analysis.

The TU6_Δ*tku70* and Δ*azf1* strains were grown on plates containing MEX medium. After 7 days, the spores were resuspended in phosphate-buffered saline (PBS) at a concentration of 1 × 10^5^/ml, and the cell suspensions were analyzed by flow cytometry (Guava easyCyte; Guava Technologies/Millipore, Hayward, CA, USA). The cell size and cellular complexity were evaluated using Express Pro Blue software and forward scatter (FSC-H) and side scatter (SSC-H) parameters, respectively.

### Enzymatic assay.

Enzymatic activity analysis was performed with the supernatant of the TU6 and Δ*azf1* strains obtained after 24 h of growth in sugarcane bagasse, pregrown in glycerol. Carboxymethylcellulase (CMCase) activity was determined by adding 30 μl of 1% carboxymethyl cellulose (CMC) prepared in sodium acetate buffer (pH 4.8) and 30 μl of the sample. The reaction mixture was incubated at 50°C for 30 min. Then, 60 μl of dinitrosalicyclic acid (DNS) was added to the reaction mixture and the mixture was heated at 95°C for 5 min. For analysis of xylanase activity, 25 μl of sample was incubated with 50 μl of 1% xylan and incubated at 50°C for 30 min. Then, 75 μl of DNS was added to the reaction mixture and heated at 95°C for 5 min. For both CMCase and xylanase activities, samples were read at an absorbance value of 540 nm. β-Glucosidase activity was determined by the addition of 50 μl of 50 mM sodium acetate buffer (pH 5.5), 10 μl of the sample, and 40 μl of 5 mM *p*-nitrophenyl (PNP)-glucoside substrate. The reaction mixture was incubated at 50°C for 15 min, followed by the addition of 100 μl of 1 M sodium carbonate. For analysis of β-xylosidase activity, 50 μl of 50 mM sodium acetate (pH 4.8) was incubated with 10 μl of the sample and 40 μl of 5 mM PNP-xyloside. The reaction mixture was incubated at 50°C for 15 min, followed by the addition of 100 μl of 1 M sodium carbonate. For analysis of cellobiohydrolase activity, 50 μl of 50 mM sodium citrate (pH 4.8), was incubated with 10 μl of the sample and 40 μl of 5 mM PNP-cellobioside. The reaction mixture was incubated at 50°C for 3 h 30 min, followed by the addition of 100 μl of 1 M sodium carbonate. For analysis of β-glucosidase, β-xylosidase, and cellobiohydrolase activity, samples were read at absorbance of 405 nm. One enzyme unit was defined as the amount of enzyme capable of releasing 1 μmol of reducing sugar per min (32). Statistical tests were performed using one-way analysis of variance (ANOVA) followed by Bonferroni's test (available in Prism software v. 5.0) for comparing the enzymatic activity levels of the parental and mutant strains.

### Quantitative real-time PCR (qRT-PCR).

T. reesei mycelia were macerated, and the RNA was extracted using TRIzol reagent (Life Technologies) according to the manufacturer's instructions. For synthesis of cDNA, 1 μg of RNA was first treated with DNase I (Fermentas) to remove genomic DNA. After this step, cDNAs were synthesized using a RevertAid H Minus first-strand cDNA synthesis kit (Thermo Scientific) according to the manufacturer's instructions. They were diluted 50× and used for qRT-PCR analysis in Bio-Rad CFX96TM equipment together with SsoFast EvaGreen Supermix (Bio-Rad), according to the manufacturer's instructions. The actin gene was used as the endogenous control. The amplification program used in this study was as follows: 95°C for 10 min followed by 39 cycles of 95°C for 10 s and 60°C for 30 s followed by a dissociation curve of 60°C to 95°C with an increment of 0.5°C for 10 s per increment. Gene expression levels were calculated as described previously (33). The primers used for amplification of identified genes are described in Table S1. MeV 4.8.1 software was used to construct the heat maps, showing the differences between the analyzed strains in the levels of gene expression. Statistical tests were performed using one-way ANOVA (and nonparametric testing), followed by Bonferroni's test (to compare all pairs of columns) (available in Prism software v. 5.0) for comparing the gene expression levels of the parental and mutant strains.

### RNA-seq analysis.

For the RNA-seq experiment, 10^6^ spores/ml of the TU6 and Δ*azf1* strains were first grown in Mandels-Andreotti medium with 1% glycerol for 24 h and then transferred to the same media containing 1% sugarcane bagasse as the sole carbon source. Once the mycelium was obtained, total RNA was extracted by the TRIzol method (Life Technologies) according to the manufacturer's instructions. Then, RNA was treated with the DNase I from Sigma-Aldrich and subsequently purified using an RNeasy minikit (Qiagen). The RNA sequencing was performed using a NextSeq system (Illumina). All the experiments corresponding to the different conditions were executed in triplicate. For gene expression analysis, quality checking of reads was performed by using FastQC (https://www.bioinformatics.babraham.ac.uk/projects/fastqc/). For the analysis of the sequences, we trimmed all reads using Trimmometic software (34). The reads were aligned with the genome and then quantified with pseudocounts using Kallisto (35). The differential expression analysis was done using DESeq2 (36). Finally, genes whose adjusted *P* values (adjpval) were lower than 0.05 and whose log2-fold change values were lower than −1 or higher than 1 were selected as differentially expressed.

### Chromatin immunoprecipitation coupled to qPCR (ChIP-qPCR).

The WT and Δ*azf1* strains were grown in 100 ml of Mandels-Andreotti medium supplemented with 1% glycerol for 24 h and then transferred to 100 ml of 1% cellulose for 12 h or 2% glucose for 48 h at 28°C and 180 rpm. Samples were cross-linked with 1% (vol/vol) formaldehyde for 20 min at room temperature before formaldehyde was quenched with 10 ml of 2.5 M glycine for 10 min at room temperature. Mycelia were ground to a fine powder under liquid N_2_ conditions and resuspended in ChiP lysis buffer (CLB) as described previously (27). Samples were sonicated for two cycles of 10 min (30 sec on and 30 sec off) before being centrifuged at 10,000 × g for 5 min at 4°C. Supernatants were collected, and protein concentrations were measured by Bradford assay (Bio-Rad) according to manufacturer’s instructions. Immunoprecipitations (IP) were carried out by first ligating 5 μg of anti-AZF1 monoclonal antibody (Rheabiotech) diluted in 200 μl of 1× PBS (phosphate-buffered saline)–0.01% Tween 20 to 20 μl of magnetic Dynabeads protein A (Invitrogen) for 10 min at room temperature. Subsequently, beads were washed once with 200 μl PBS-Tween before being resuspended in 50 μl CLB. A total of 2.2 mg of protein was added to the beads for each sample, and the volume was adjusted to 1 ml with CLB. Protein samples and beads were incubated at 4°C with rotation for 2 h before beads were washed as described previously (27). Sample elution and reverse cross-linking were carried out as described previously (27, 37). DNA was treated with RNase A at a concentration of 1 mg/ml at 37°C for 1 h and purified by the use of a PCR purification kit (Illustra GFX PCR DNA purification kit; GE Healthcare), according to the manufacturer’s instructions. Subsequently, 1 μl of DNA was used for qPCR in Bio-Rad CFX96TM equipment using SsoFast EvaGreen Supermix (Bio-Rad), according to the manufacturer's instructions. The amplification program used in this study was as follows: 95°C for 10 min followed by 39 cycles of 95°C for 10 s and 60°C for 30 s followed by a dissociation curve of 60°C to 95°C with an increment of 0.5°C for 10 s per increment. Primers used in the qPCR are described in Table S1. DNA enrichment at each gene promoter site was calculated using the percent input method (37), and positive controls included cross-linked but not immunoprecipitated samples (= input). All experiments were carried out in biological triplicate. The tests of statistical significance were performed using one-way (nonparametric) ANOVA followed by the Bonferroni test (comparing all pairs of columns; available in Prism software v.8.0). Significance is indicated in the form of probability values (*, *P* < 0.05; **, *P* < 0.01; ***, *P* < 0.001; ****, *P* < 0.0001).
