# Supplementary figures and images for: A Novel Cys2His2 Zinc Finger Homolog of AZF1 Modulates Holocellulase Expression in Trichoderma reesei
Source: mSystems. 2019 Jun 18;4(4):e00161-19. doi: 10.1128/mSystems.00161-19 (PMC6581689; doi:10.1128/mSystems.00161-19)

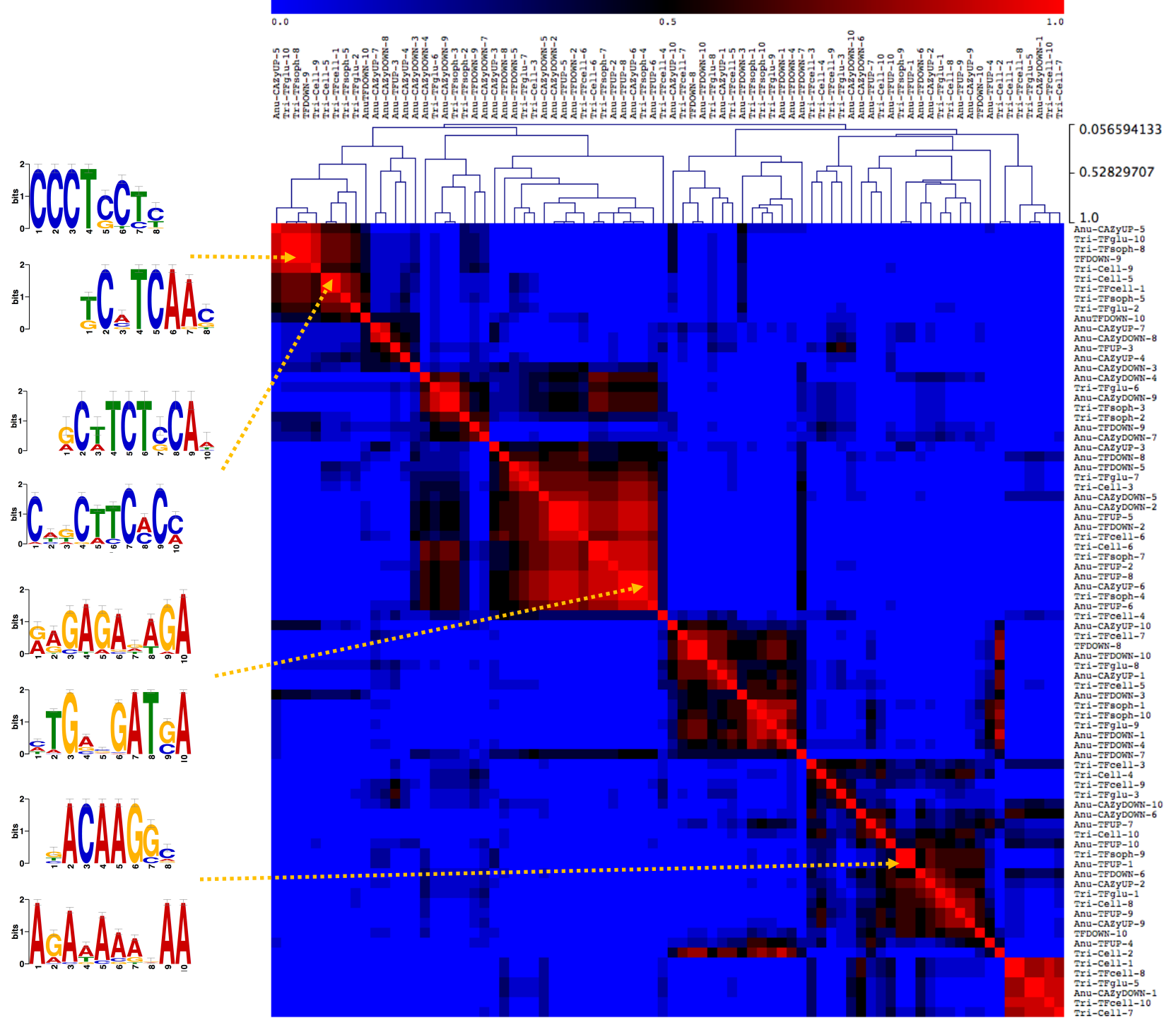

Supplement: FIG S1 [file mSystems.00161-19-sf001.pdf]

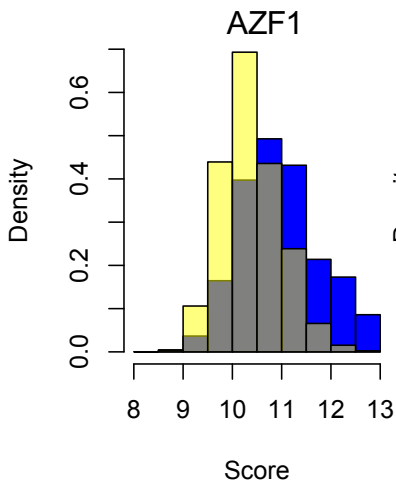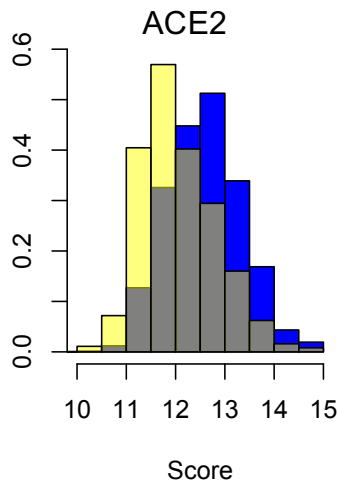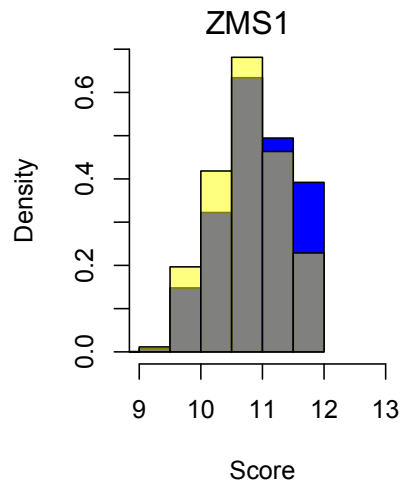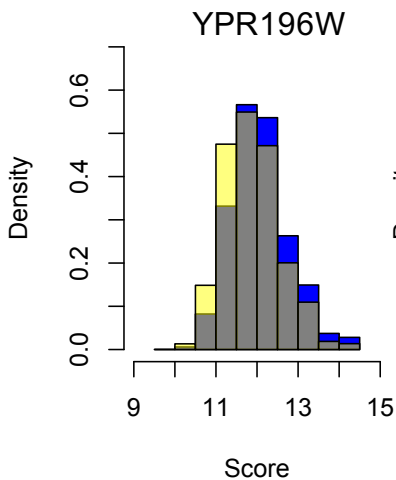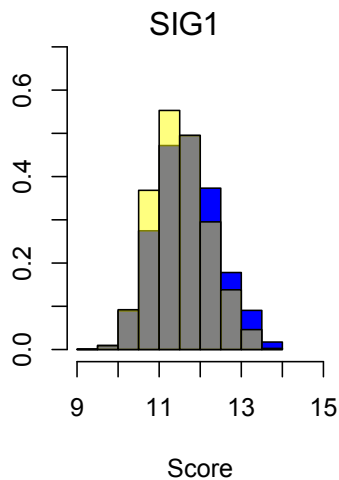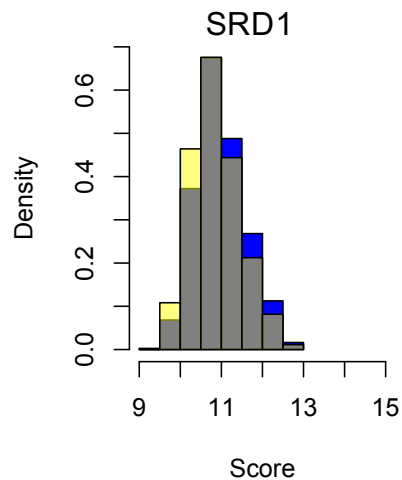

Supplement: FIG S2 [file mSystems.00161-19-sf002.pdf]

TBF1

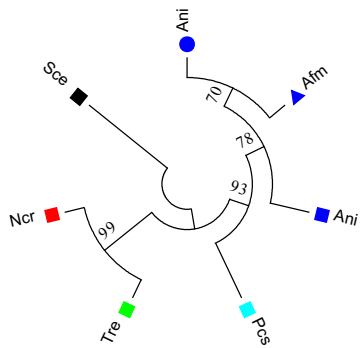

Azf1

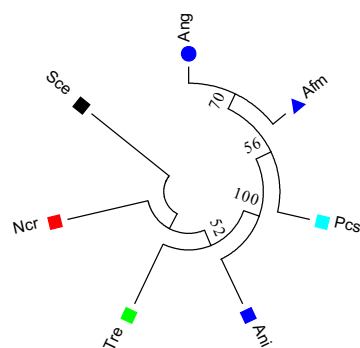

ACE2

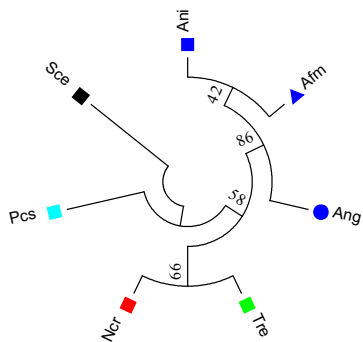

CUP2

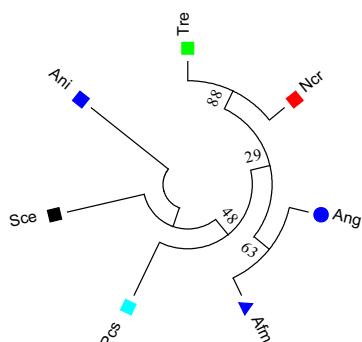

YPR196W

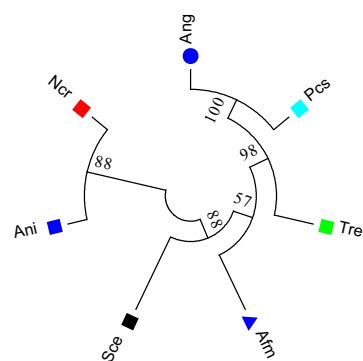

ZMS1

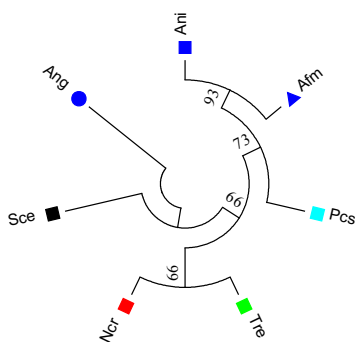

SRD1

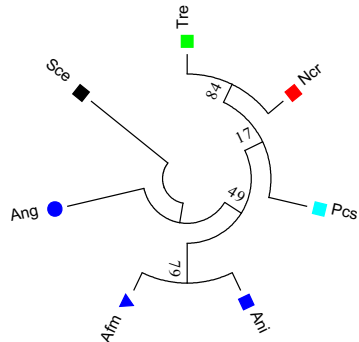

Supplement: FIG S3 [file mSystems.00161-19-sf003.pdf]

A

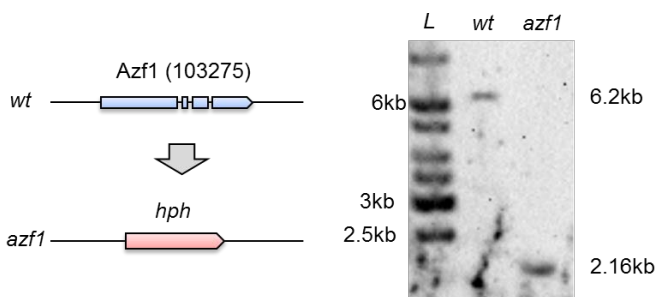

B

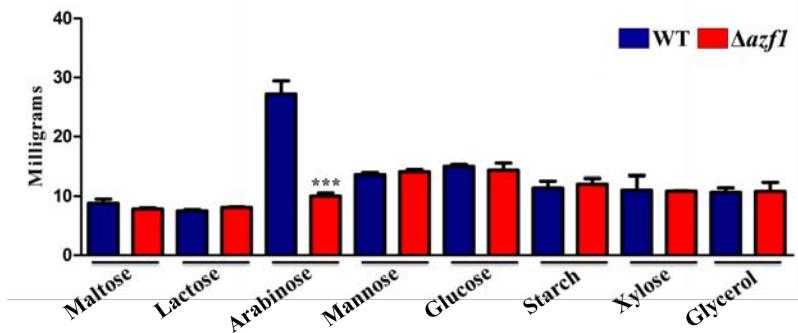

C

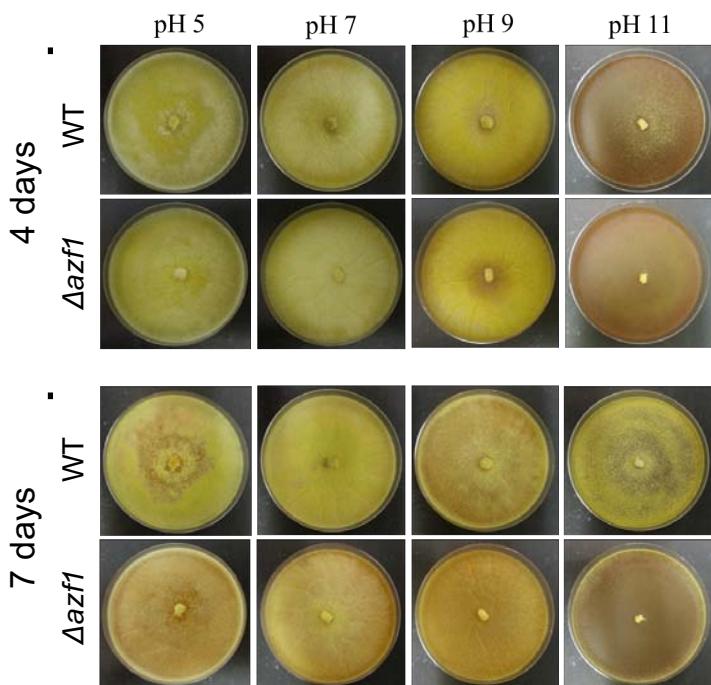

Supplement: FIG S4 [file mSystems.00161-19-sf004.pdf]

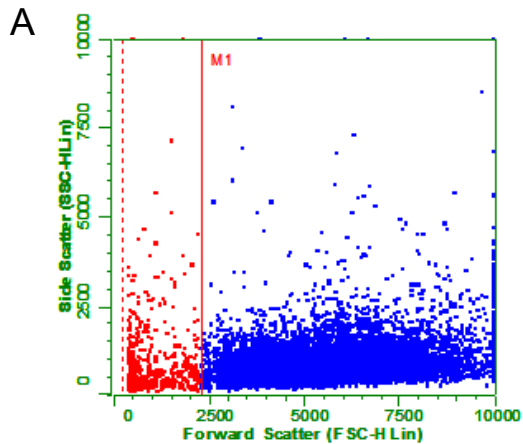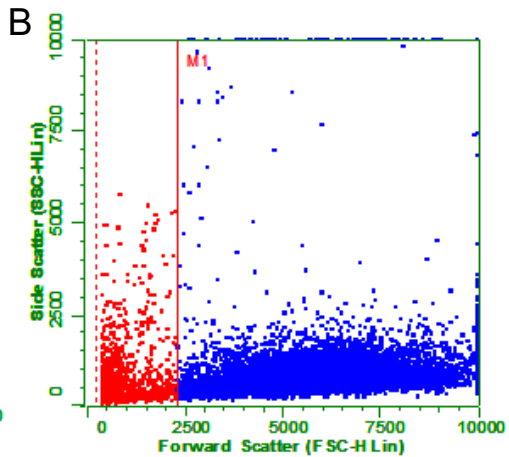

**C**

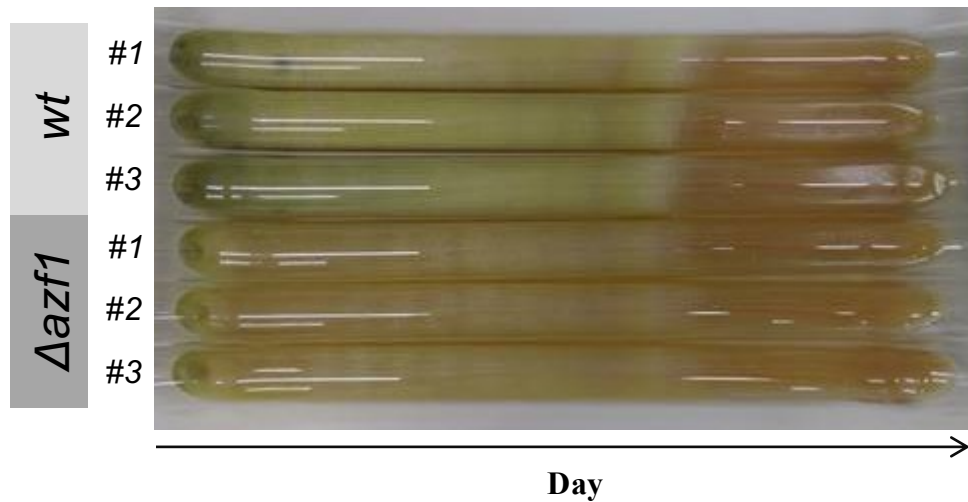

Supplement: FIG S5 [file mSystems.00161-19-sf005.pdf]

A

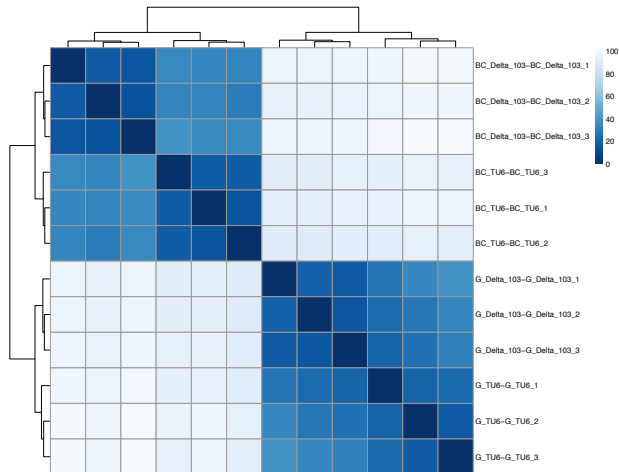

B

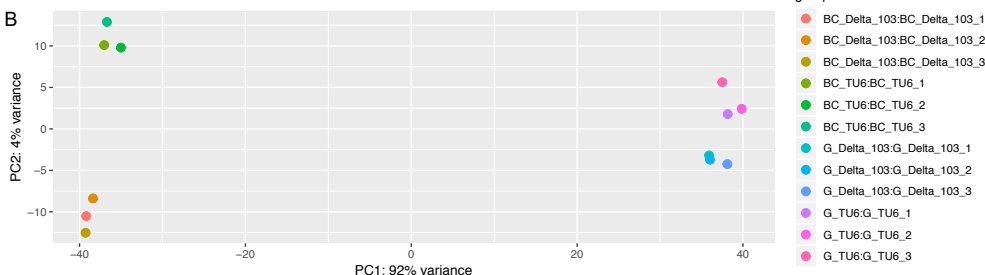

C

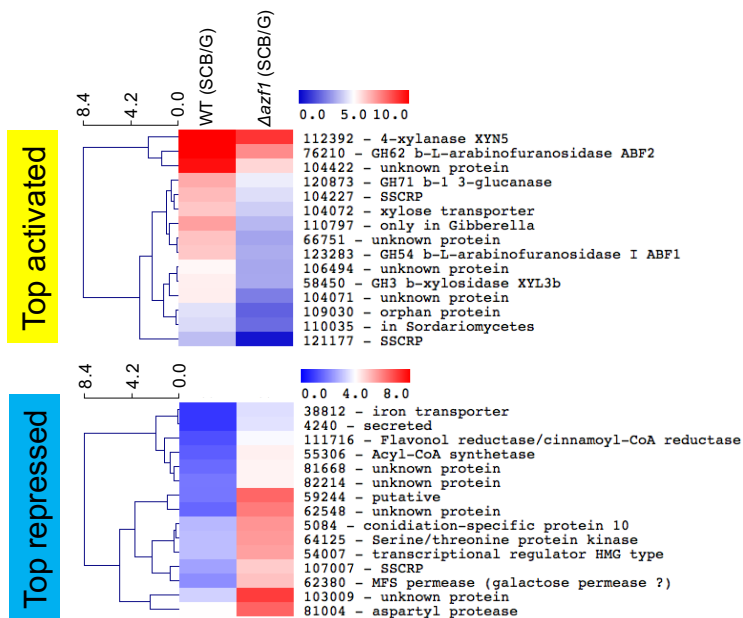

Supplement: FIG S6 [file mSystems.00161-19-sf006.pdf]

A

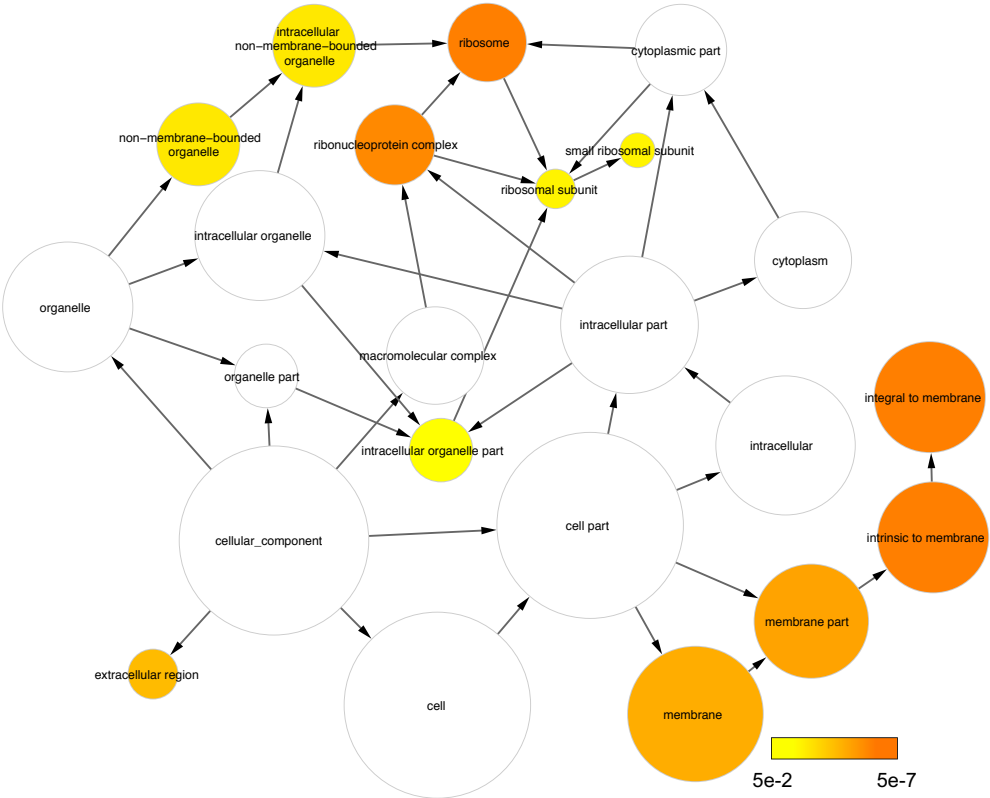

B

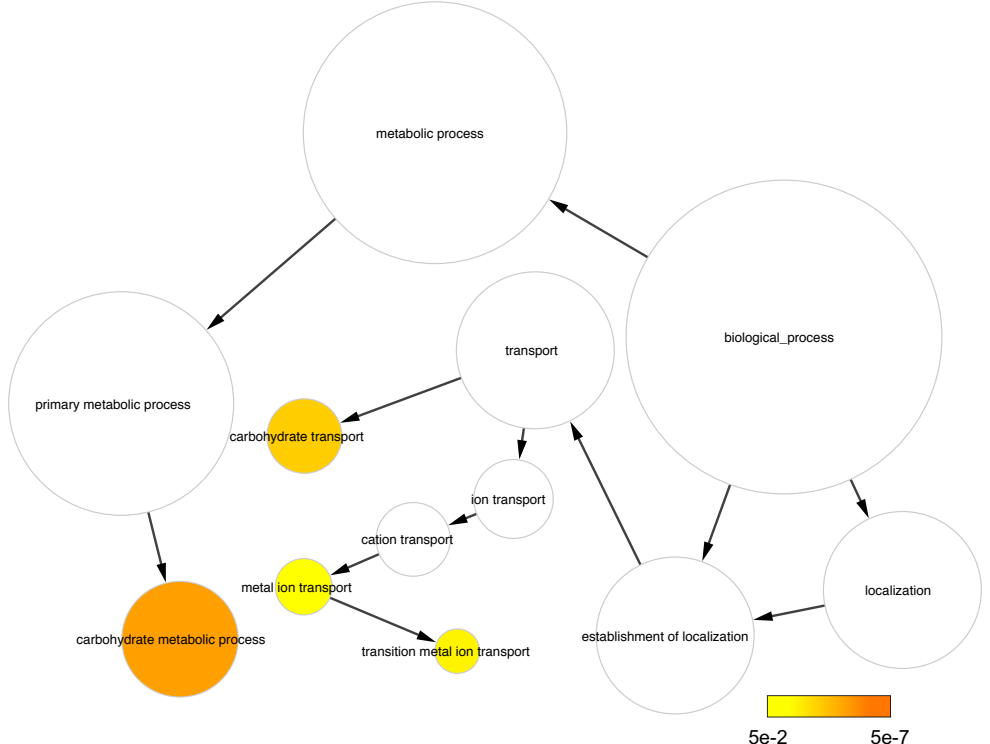

Supplement: FIG S7 [file mSystems.00161-19-sf007.pdf]
