# Supplementary material for: A Novel Cys2His2 Zinc Finger Homolog of AZF1 Modulates Holocellulase Expression in Trichoderma reesei
Source: mSystems. 2019 Jun 18;4(4):e00161-19. doi: 10.1128/mSystems.00161-19 (PMC6581689; doi:10.1128/mSystems.00161-19)
Supplement: TABLE S1 [file mSystems.00161-19-st001.docx]

| Organisms/Datasets | | Genes | |
| --- | --- | --- | --- |
| *Trichoderma reesei* | |  | |
| TF glu | | 119759, 21270, 78049, 75472, 74346, 57676, 74252, 54437, 66047, 55759, 57534, 102497, 102499, 109394, 104182, 112202, 112499, 105520 | |
| TF soph | | 73654, 67418, 120224, 120428, 120908, 80200, 62244, 65746, 66828, 68455, 55274, 70351, 58389, 21997, 123881, 121107, 73792, 72611 | |
| TF cell | | 110152, 120698, 108775, 68254, 69972, 121164, 105269 | |
| Cellulases | | 72567, 76672, 123989, 123992, 120749, 73643, 49976, 122081, 22197, 121735, 82227, 120312, 82616, 123232, 120961, 46816, 76227, 49081, 74223, 123818, 120229, 111849 | |
| *Aspergillus nidulans* | |  | |
| CAZyUP | | AN5176, AN1818, AN3613, AN9380, AN0245, AN5309, AN7396, AN5282, AN3418, AN1285, AN6093, AN2828, AN0494, AN5267, AN8953, AN5463, AN1428, AN6470, AN1273, AN1870, AN4825, AN0012, AN3860, AN1477, AN3903, AN7908, AN1502, AN2632, AN6428, AN9390, AN3358, AN5320, AN0787, AN7349, AN2325, AN2528, AN2388, AN2395, AN9276, AN6518, AN7307, AN4871, AN2569, AN3388, AN11143, AN6106, AN0472, AN9340, AN2017, AN2425, AN4102, AN3368, AN3402, AN3370, AN8241, AN0452, AN7646, AN0741, AN5727, AN6464, AN7413, AN3733, AN3297, AN8481, AN7275, AN9069, AN3925, AN9365, AN1277, AN8007, AN1197, AN0299, AN3049, AN2936, AN7505, AN1015, AN3013, AN2953, AN2303, AN3837, AN2463, AN4601, AN7715, AN1811, AN1416, AN4566, AN0712, AN4860 | |
| CAZyDown | | AN2534, AN5748, AN4686, AN5663, AN5635, AN7301, AN3949, AN6857, AN3588, AN3354, AN0160, AN3308, AN7158, AN5902, AN3730, AN6352, AN8444, AN3309, AN8606, AN3566, AN8421, AN6405, AN4372, AN8135, AN8314, AN3307, AN0487, AN6460 | |
| TF Up | | AN1812, AN1414, AN3369, AN8902, AN9397, AN9373, AN3356, AN3675, AN7553, AN7190, AN10059, AN10789, AN4197, AN0096, AN7610, AN1402, AN11753, AN8298, AN5775, AN8978, AN2650, AN6858, AN2290, AN0680, AN6846, AN5924, AN2276, AN5349, AN2782, AN5870, AN7734, AN7050, AN0863, AN1368, AN0835, AN6884, AN10295, AN0689, AN4035, AN2597, AN5808, AN2763, AN7942, AN2270, AN10638, AN3683, AN10334, AN1077, AN3650, AN1406, AN1705, AN7560, AN3986, AN8177 | |
| TF Down | | AN0748, AN9221, AN4933, AN5910, AN0094, AN5894, AN5220, AN6221, AN0937, AN7346, AN3637, AN10378, AN4744, AN8431, AN3224, AN5752, AN10970, AN11185 | |
|  | |  | |
| Protein ID | **Gene** | **5’→3’ (Foward)** | **5’→3’ (Reverse)** |
| 72567 | *cel6a* | ACAAGAATGCATCGTCTCCG | TGTTCCACCCGTTGTAGTTG |
| 76672 | *cel3a* | CTGTACATCACCTACCCATC | TAGCTGAGATCTCGTCGTC |
| 123989 | *cel7a* | CCGAGCTTGGTAGTTACTCTG | GGTAGCCTTCTTGACTGAGT |
| 123992 | *swo* | GAGTGAATGTCTTGATGG | CCAAACTATACGAGTAGCC |
| 120749 | *cel1a* | TTTGCCTGGTCGCTCATG | AATCAGCTCGTCAAACAGCG |
| 73643 | *cel61a* | GCGCCACTGTTCCTGGAG | ACCGCTGCCACCACACTG |
| 49976 | *cel45a* | CAGCGACGTCTACATTGG | TGGTCCAGAATGCACTCG |
| 122081 | *cel7b* | CCCTCAACACTAGCCACCAG | AGGTCTTGGAGGTGTCAACG |
| 22197 | *cel1b* | CCATCTACATCACCGAGAACG | TCCAAGTGCGAGTCAAAGTAG |
| 121735 | *cel3b* | CCAGGATAACTTCAACGAGGG | ATGTGGAGGTTGGAGAACTTG |
| 82227 | *cel3c* | GCGTACAATGGCATCAATGG | ATCCCAACCCCATTCCTTTC |
| 120312 | *cel5a* | GCCACTACTATCACCACTTCG | GTACAGCCAAAGTCAAAACCC |
| 82616 | *cel5b* | AAAGTACCGTCCAACACCC | TGGTAACAGCATTGGGAGTG |
| 123232 | *cel12a* | AGATTGCCATTCCCCAGAAG | CCCGAGTACGTGACATGATTC |
| 120961 | *cel61b* | ACTATGTCTTCCGCCATGAAC | CACGCACTGAGGATAGTTCTG |
| 46816 | *cel3d* | AACCCAGCATATCTCAACTACG | CTTGAAGGTAGCGTAAGACAGG |
| 76227 | *cel3e* | ATGTCTGGAAGTGAGGTTGC | TCGTGAGTCCAAAGTGAACAG |
| 49081 | *cel74a* | GCCTTGTATCTGACCTATTCCG | TGATGTCTTTCCAAGTTCCCC |
| 74223 | *xyn1* | GGCCAAATTATCGTCAACTGTC | TCTGTCTTTTGGGCTTGGAG |
| 123818 | *xyn2* | TGTCAACGAGCCTTCCATC | TCTGCACAGTAACAGTTCCG |
| 120229 | *xyn3* | AAGTCATCCGCACCCATG | GTTCAAAACTCACCCAAGCAC |
| 111849 | *xyn4* | TGTCAGCAATTCGGGTCTTC | GACAGGTTGGCAAAATGGTG |
| 103275 | *azf1* | AGAATCTCACCATACGCCATG | CAATGCCCATGTGAAAGACG |
| 44504 | *actin* | TGAGAGCGGTGGTATCCACG | GGTACCACCAGACATGACAATGTT |
| Chipcel7a | | GTCCAATCTCAGCTGGTGAT | ACTGCCCTTGGTTGTATGCA |
| Chipswo | | AGCAGCAGCGGCAATAACAA | GCTACATCTGTGGATAGGTAG |
| Chipcel45a | | CTTCGTCAACAGACTCTCTC | GTGCTGTATGTGGGAGTCTA |
