# Supplementary material for: A Novel Cys2His2 Zinc Finger Homolog of AZF1 Modulates Holocellulase Expression in Trichoderma reesei
Source: mSystems. 2019 Jun 18;4(4):e00161-19. doi: 10.1128/mSystems.00161-19 (PMC6581689; doi:10.1128/mSystems.00161-19)
Supplement: TABLE S2 [file mSystems.00161-19-st002.docx]

| Name | Protein ID | Start | End | Strand | Score | p-val | Sequence | Diffential-Expresison |
| --- | --- | --- | --- | --- | --- | --- | --- | --- |
| Cel7a | 123989 | 467 | 474 | + | 12.67 | 3.96e-05 | AAGAAGAC | yes |
| swo | 123992 | 476 | 469 | - | 12.17 | 5.75e-05 | AAGCAGAA | yes |
| Cel1a | 120749 | 929 | 936 | + | 14.06 | 2.18e-05 | AAGAAGAA | yes |
| Cel61a | 73643 | 820 | 813 | - | 14.06 | 2.18e-05 | AAGAAGAA | yes |
| Cel45a | 49976 | 954 | 947 | - | 14.06 | 2.18e-05 | AAGAAGAA |  |
| Cel7b | 122081 | 67 | 60 | - | 11.67 | 9.33e-05 | AGGAAGAA | yes |
| Cel1b | 122197 | 946 | 939 | - | 14.06 | 2.18e-05 | AAGAAGAA |  |
| Cel3b | 121735 | 251 | 244 | - | 14.06 | 2.18e-05 | AAGAAGAA | yes |
| Cel5a | 120312 | 822 | 829 | + | 14.06 | 2.18e-05 | AAGAAGAA | yes |
| Cel5b | 82616 | 104 | 111 | + | 11.67 | 9.33e-05 | GAGAAGAA |  |
| Cel3d | 46816 | 367 | 360 | - | 14.06 | 2.18e-05 | AAGAAGAA |  |
| Cel3e | 76227 | 458 | 465 | + | 14.06 | 2.18e-05 | AAGAAGAA |  |
| Cel74a | 49081 | 317 | 324 | + | 11.67 | 9.33e-05 | GAGAAGAA | yes |
| xyn1 | 74223 | 925 | 932 | + | 12.67 | 3.96e-05 | AAGAAGAC |  |
| xyn2 | 123818 | 746 | 739 | - | 11.67 | 9.33e-05 | GAGAAGAA |  |
| xyn3 | 120229 | 409 | 402 | - | 12.67 | 3.96e-05 | AAGAAGAC |  |
| xyn4 | 111849 | 288 | 295 | + | 12.67 | 5.75e-05 | AAGCAGAA |  |
